# The physiological determinants of low-level urine cadmium: an assessment in a cross-sectional study among schoolchildren

**DOI:** 10.1186/s12940-017-0306-5

**Published:** 2017-09-12

**Authors:** Hongyu Wang, Xavier Dumont, Vincent Haufroid, Alfred Bernard

**Affiliations:** Louvain Centre for Toxicology and Applied Pharmacology, Institut de Recherche Expérimentale et Clinique (IREC), Université catholique de Louvain, Avenue Emmanuel Mounier 53.02, B-1200 Brussels, Belgium

**Keywords:** Cadmium, Biomarker, Club cell protein, alfa_1_-microglobulin, Protein HC, Retinol-binding protein, β_2_-microglobulin

## Abstract

**Background:**

Recent studies in children have reported associations of urinary cadmium (U-Cd), used as biomarker of Cd body burden, with renal dysfunction, retarded growth and impaired cognitive development in children. Little is known, however, about factors influencing U-Cd in children and likely to act as confounders.

**Methods:**

In a cross-sectional study involving 249 schoolchildren (mean age, 5.72 years; 138 boys), we measured the urine concentrations of cadmium, zinc, lead, albumin, alpha_1_-microglobulin (A1M), retinol-binding protein, β_2_-microglobulin and club cell protein (CC16). Determinants of U-Cd expressed per creatinine or adjusted to specific gravity were identified by multiple regression analyses.

**Results:**

Girls and boys had similar median concentrations of U-Cd (0.22 and 0.24 μg/L, 0.33 and 0.35 μg/g creatinine, respectively). When models were run without including creatinine or specific gravity among independent variables, urinary zinc, urinary A1M and age emerged as the strongest predictors of U-Cd expressed per g creatinine or adjusted to SG. When adding creatinine among predictors, urinary creatinine emerged as an additional strong predictor correlating negatively with U-Cd per g creatinine. This strong residual influence of diuresis, not seen when adding specific gravity among predictors, linked U-Cd to U-A1M or U-CC16 through secondary associations mimicking those induced by Cd nephrotoxity.

**Conclusions:**

In young children U-Cd largely varies with diuresis, zinc metabolism and urinary A1M. These physiological determinants, unrelated to Cd body burden, may confound the child renal and developmental outcomes associated with low-level U-Cd.

## Background

Cadmium (Cd) is a highly toxic and cumulative metal that after long term exposure can cause serious health effects, including renal dysfunction, bone demineralization and by inhalation lung cancer. Diet and tobacco smoke are the main sources of human exposure to Cd [1]. When absorbed by inhalation or ingestion, Cd accumulates over lifetime in the body, especially in the kidneys, with a biological half-life of more than 15 years [2]. The kidney, the main site of Cd storage, is generally considered to be also the critical target organ i.e. the first organ to be damaged after prolonged exposure. The earliest nephrotoxic effect of Cd is a dysfunction of the proximal tubule, resulting in an increased urinary excretion of low-molecular-weight (LMW) proteins, such as retinol-binding protein, alpha_1_-microglobulin or β_2_-microglobulin [3, 4]. This LMW proteinuria, also referred to as tubular proteinuria, is due to the decreased reabsorption capacity of defective proximal tubular cells [3].

An important concept in Cd risk assessment is the assumption that U-Cd is a reliable non-invasive measure of the amount of metal stored in the body. As explained elsewhere [1], international regulatory bodies recently endorsed this concept when establishing the tolerable dietary intakes or occupational exposures to Cd. The vast majority of epidemiological studies also relied on this concept when implicating low-level Cd exposure as a risk factor for bone, cardiovascular and other degenerative diseases [1]. In these studies, the use of U-Cd as indicator of Cd body burden is an argument for excluding the possibility of reverse causation since in most cases the studied outcomes (e.g. renal or developmental effects) are unlikely to increase the body burden of the heavy metal. Of concern, recent research suggests that Cd can exert its toxicity during the first years of life and this at the exposure levels prevailing in most industrialized countries. Several studies among children with low dietary exposure to Cd have indeed associated an increase of U-Cd with renal dysfunction (decreased glomerular filtration rate and increased proteinuria), retarded growth and impaired cognitive development (learning disability, special education utilization, cognitive delays) [5–10].

However, the significance of U-Cd as an index of cumulative exposure to the metal is now called into question by studies revealing that low-level U-Cd of adults or adolescents is predominantly influenced by factors unrelated to Cd body burden such as recent exposure, urinary flow or the co-excretion of Cd with urinary proteins [11, 12–18]. Particularly challenging is the finding that children have U-Cd values comparable to those of adults despite a Cd body burden about ten times lower [18]. These findings raise doubt about the significance of low-level U-Cd in children and therefore about the significance of associations seen with this exposure measure. Therefore, the purpose of this study was to investigate the physiological and environmental factors that influence U-Cd levels in children with low background exposure to the heavy metal.

## Methods

### Study population

Study participants were 249 children (138 boys, mean age, 5.75 years) in the third year of kindergarten. These children were recruited from 30 schools located in Belgium in the framework of an epidemiological study investigating the effects of various environmental stressors on child’s health. The origin of children, the recruitment protocol and the participation rate are described in detail elsewhere [18]. Children participated to the study with their assent and the informed consent of their parents. A parent self-administered questionnaire was used to obtain information about children health and factors likely to impact on kidney function or to be sources of Cd exposure. Examinations of children, which took place in schools, included the measurement of body weight and height and the collection of an untimed urine sample. Children were examined between 9:00 A.M. and 3:00 P.M., and in most cases (*n* = 219) urine was collected before noon. Samples of urine were collected in Cd-free containers and stored at −20 °C until analysis. The study population did not include seven children who were removed because their U-Cd (*n* = 3) or urinary creatinine (*n* = 4) deviated by more than three geometric SDs from the geometric mean in the initial population. There were no reports of diabetes or renal disease among study participants. The Ethics Committee of the Faculty of Medicine of the Catholic University of Louvain approved the study protocol that complied with applicable requirements of international regulations.

### Analytical methods

We measured Cd, Pb and Zn in urine by inductively coupled argon plasma mass spectrometry (ICP-MS) with an Agilent 7500 instrument (Agilent Technologies. Santa Clara, CA, USA), as described in a previous study [19]. Briefly, urine specimens (500 μl) were diluted quantitatively [1 + 9 (vol/vol)] with a 1% nitric acid/0.5% hydrochloric acid solution containing scandium, germanium, rhodium and iridium as internal standards. As described elsewhere [17] our Cd analyses by ICP-MS were unaffected by the interference from molybdenum. The detection and quantification limits were respectively 0.02 and 0.05 μg/L for Cd, 0.03 and 0.09 μg/L for Pb and 0.6 and 1.8 μg/L for Zn. The accuracy of our method for Cd measurement was ascertained by the participation to the University of Erlangen quality assurance program. For the periods of 2011–2014 corresponding to the measurements performed in this study, the results of U-Cd (μg/L) vs. the reference value were as follow: low U-Cd, 0.21 vs. 0.22, 0.30 vs. 0.30, 0.25 vs. 0.22, 0.29 vs. 0.25, 0.20 vs. 0.19; high U-Cd, 0.69 vs. 0.65, 0.78 vs. 0.81, 0.50 vs. 0.47, 0.71 vs. 0.65, 0.61 vs. 0.60. The compliance with reference values averaged 106% (SD, 8.7) for low values and 104% (SD, 5.1) for high values of U-Cd. A similar compliance with references values was obtained for the determination of Pb and Zn (results not shown). The urinary concentrations of β_2_-microglobulin (U-β_2_m), alpha_1_-microglobulin (U-A1M), club cell protein (U-CC16), retinol-binding protein (U-RBP) and albumin (U-Alb) were determined by automated latex immunoassays using Dakopatts antibodies and standards based on commercially available proteins or on proteins purified in our laboratory [20–23]. Because of insufficient urine volume, we could not measure U-β_2_m in 11 samples, U-CC16 in 50 samples and urinary lead (U-Pb) in one sample. We also excluded from the analyses of U-CC16 another 17 samples with undetectable values even though including them with the immunoassay detection limit yielded the same pattern of significant associations. Creatinine in urine (U-Creat) was determined by a modified Jaffé reaction using a Beckman Synchron LX 20 analyser (Beckman Coulter GmbH, Krefeld, Germany) [24]. Specific gravity of urine (SG) was determined with a refractometer and concentrations were transformed to the mean value of urinary density in the studied group (1.021 g/mL) by using the formula: C_SG_ = Cm × 0.021/(SG − 1.000) where C_SG_ is the adjusted value for SG and Cm is the measured concentration [25]. The laboratory is ISO15189 certified for the measurement of 20 trace elements in urine, including Cd, Zn and Pb.

### Statistical analyses

Data analyses were performed using version 12 of the JMP (SAS Institute Inc., Cary, NC, USA). All characteristics and biological parameters in urine were described as median with interquartile range (IQR) and were log-transformed to approximate normal distribution. To adjust for variations in urine dilution, urinary biomarkers were expressed per g of creatinine or adjusted to U-SG. Student’s t-test was used to assess gender differences with regard to biomarkers and their potential predictors. Associations between variables were evaluated by Pearson’s correlation analysis. Determinants of U-Cd were assessed by backward stepwise regression analyses testing as potential predictors age, gender, parental smoking, body mass index (BMI), time of urine collection, urinary zinc (U-Zn), U-Alb, a LMW protein in urine (U-RBP, U-β_2−_m, U-A1M or U-CC16). We run these models by testing five methods to account for the influence of diuresis. In the first method, the urinary concentrations of heavy metals and proteins were expressed per g of creatinine. In the second method, metals and proteins in urine were also expressed per g of creatinine but we added U-Creat as a separate independent variable to remove the possible residual influence of diuresis as evaluated by U-Creat. In the third method, we expressed metals and proteins in urine per liter and tested U-Creat as a separate independent variable as recommended by Barr et al. [26]. In the fourth method, urinary metals and proteins were adjusted to SG. In the fifth method, we expressed metals and proteins in urine per liter and we added U-SG as a separate independent variable. We optimized these models by minimizing the Akaike information criterion. To further explore the confounding effect of diuresis, we compared by ANOVA with the Dunnett’s post-hoc test the urinary excretion of LMW proteins across quartiles of increasing U-Cd expressed per g of creatinine, without and with further adjusting these biomarkers for their residual association with U-Creat. All *P*-values were two-sided with the level of statistical significance at *P* < 0.05.

## Results

Characteristics of children and the concentrations of metals in urine are summarized in Table 1. The mean age of the studied group was 5.72 years and 55.4% of them were boys. Boys had significantly higher U-Creat and U-SG than girls. Boys also had higher U-Pb but this difference disappeared after adjustment with creatinine or SG. There were, by contrast, no gender differences in U-Cd and U-Zn whatever the method used for urine dilution adjustment. As displayed in Table 2, the two sexes had also very similar levels of U-RBP, U-β_2_m and U-CC16. Girls, however, had higher U-Alb than boys while their U-A1M was lower.

**Table 1:** Characteristics of children and concentrations of metals in urine

	Girls	Boys	*P*-value
N^a^	111	138	
Age (years)	5.83 (5.5–6.0)	5.75 (5.42–5.92)	0.07
BMI (kg/m^2^)	15.4 (14.6–17.0)	15.8 (15.1–17.0)	0.49
U-Creat (g/L)	0.64 (0.43–0.91)	0.73 (0.57–0.90)	0.02
U-SG	1.021 (1.016–1.025)	1.023 (1.020–1.025)	0.01
U-Cd			
μg/L	0.22 (0.16–0.31)	0.24 (0.18–0.30)	0.12
μg/g creatinine	0.35 (0.27–0.47)	0.33 (0.26–0.45)	0.43
μg/L adjusted for SG	0.23 (0.19–0.31)	0.22 (0.17–0.29)	0.49
U-Zn			
μg/L	330 (178–475)	347 (222–481)	0.09
μg/g creatinine	502 (348–686)	470 (350–657)	0.96
μg/L adjusted for SG	339 (229–472)	317 (235–427)	0.87
U-Pb^a^			
μg/L	0.92 (0.54–1.49)	1.08 (0.72–1.66)	0.03
μg/g creatinine	1.42 (1.04–2.00)	1.60 (1.11–2.18)	0.29
μg/L adjusted for SG	0.95 (0.65–1.51)	1.03 (0.78–1.57)	0.28

^a^Girls, *n* = 110; boys, *n* = 138. Values are median (interquartile range)

**Table 2:** Concentrations of proteins in urine

	Girls	Boys	*P*-value
U-RBP			
μg/L	104 (69.3–136)	96.2 (68.8–153)	0.56
μg/g creatinine	158 (124–201)	139 (102–200)	0.15
μg/L adjusted for SG	101 (75.8–144)	96.2 (66.8–139)	0.23
U-Alb			
mg/L	2.62 (1.26–5.59)	1.55 (0.85–3.37)	0.04
mg/g creatinine	4.10 (2.30–7.60)	2.40 (1.28–4.70)	0.002
mg/L adjusted for SG	2.65 (1.46–5.57)	1.56 (0.80–3.09)	0.004
U-A1M			
mg/L	2.10 (1.10–3.30)	2.60 (1.57–3.92)	0.003
mg/g creatinine	3.14 (1.89–5.63)	3.75 (2.53–5.38)	0.08
mg/L adjusted for SG	2.10 (1.31–3.30)	2.50 (1.60–3.93)	0.06
U-β_2_m^a^			
μg/L	77.5 (38.5–121)	82.0 (46.0–124)	0.62
μg/g creatinine	135 (86.4–182)	119 (72.8–153)	0.33
μg/L adjusted for SG	83.4 (60.4–121)	76.0 (49.5–106)	0.40
U-CC16^b^			
μg/L	1.79 (0.82–3.49)	1.50 (0.79–2.53)	0.35
μg/g creatinine	2.80 (1.10–5.70)	2.00 (1.10–4.40)	0.32
μg/L adjusted for SG	1.79 (0.80–3.61)	1.39 (0.74–2.55)	0.27

^a^Girls *n* = 104; boys, *n* = 134. ^b^Girls, *n* = 78; n = 104 boys. Values are median (interquartile range)

**Fig. 1:**
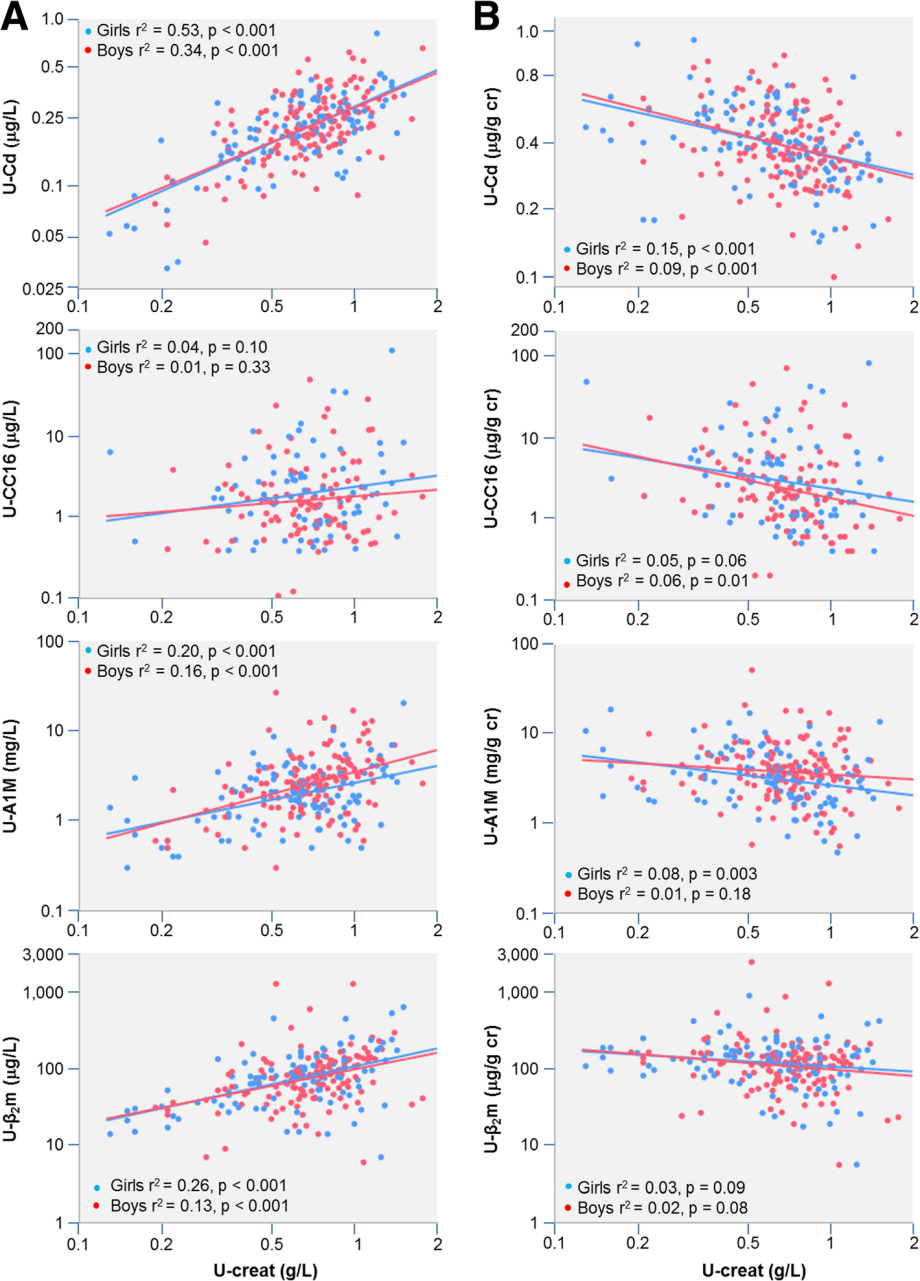
Associations of U-Cd, U-CC16, U-A1M, and U-β_2_m with urinary creatinine expressed par liter (Panel **a**) or per g of creatinine (Panel **b**)

Table 3 shows the univariate associations between heavy metals, renal biomarkers and their potential predictors for urinary biomarkers expressed per g creatinine (Table 3) or after adjustment with U-SG (Table 3). Although the values of U-SG and U-Creat were highly correlated (*r* = 0.84, *P* < 0.001), there were noticeable differences in the correlation patterns according to the method of adjustment for urine dilution. When expressed per g of creatinine, U-Cd, U-A1M, U-CC16 and U-β_2_m correlated negatively with U-Creat and in some cases even with U-SG (Table 3). This suggests, as illustrated in Fig. 1, that dividing by U-Creat does not completely abolish the associations of these urinary biomarkers with U-Creat but rather changes its direction from a positive into a negative one. Of note, there were virtually no differences in this correlation inversion between girls and boys at the exception of U-A1M, for which this phenomenon occurred mainly in girls. Such residual influence of diuresis after dividing by U-Creat was not observed with U-RBP and U-Alb, neither with U-Pb and U-Zn. Interestingly, the over-adjustment with creatinine is linked to the β coefficient of the log-log regression of the biomarker concentration per liter with U-Creat. For those biomarkers expressed per g creatinine showing no residual correlation with U-Creat, this β coefficient was close to one: U-Zn, 0.92; U-Pb, 0.95 and U-RBP, 0.94 (*r* = 0.66, 0.59 and 0.66 respectively, all *P* < 0.001). By contrast for biomarkers with a strong inverse correlation with U-Creat, this β coefficient was much lower: U-Cd, 0.71; U-A1M, 0.73; U-β_2_m, 0.43; U-CC16, 0.36 (*r* = 0.67, 0.44, 0.44 and 0.14, respectively, all *P* < 0.001 except for U-CC16, *P* = 0.06). As expected, the four LMW urinary proteins correlated with each other but none of them correlated with U-Alb. The only statistically significant correlations between the concentrations per g creatinine of the three heavy metals (U-Cd, U-Zn and U-Pb) and urinary proteins were those linking U-Cd to U-A1M or U-CC16.

**Table 3:** Pearson’s correlation coefficient between metals and proteins in urine and their potential predictors when analytes were (A) adjusted on the basis of urinary creatinine (A) or of specific gravity (B)

	Age	BMI	U-Cr	U-SG	U-Cd	U-Zn	U-Pb	U-RBP	U-Alb	U-A1M	U-CC16	U-β_2_m
(A)												
Age	1.00											
BMI	0.10	1.00										
U-Cr	0.07	0.07	1.00									
U-SG	0.07	0.07	0.84*	1.00								
U-Cd	0.12	−0.01	−0.34*	−0.16^#^	1.00							
U-Zn	0.01	−0.08	−0.07	0.02	0.28*	1.00						
U-Pb	−0.04	0.08	−0.04	0.05	0.14^$^	0.14^$^	1.00					
U-RBP	−0.06	−0.09	−0.06	−0.05	0.12	0.10	0.05	1.00				
U-Alb	−0.03	−0.03	−0.12	−0.22*	−0.10	−0.09	−0.10	0.17	1.00			
U-A1M	−0.10	−0.04	−0.18^#^	−0.10	0.18^#^	0.02	0.10	0.41*	−0.07	1.00		
U-CC16	0.03	−0.05	−0.24^#^	−0.19^$^	0.17^$^	0.02	0.08	0.37*	−0.04	0.58*	1.00	
U-β_2_m	−0.14^$^	−0.11	−0.16^$^	−0.09	0.10	0.05	0.03	0.42*	0.02	0.55*	0.30*	1.00
(B)												
Age	1.00											
BMI	0.10	1.00										
U-Cr	0.07	0.07	1.00									
U-SG	0.07	0.07	0.84*	1.00								
U-Cd	0.16^$^	0.02	0.15^$^	0.01	1.00							
U-Zn	0.03	−0.05	0.29*	0.15^$^	0.30*	1.00						
U-Pb	−0.01	0.10	0.27*	0.15^$^	0.16^$^	0.21*	1.00					
U-RBP	−0.03	−0.06	0.29*	0.08	0.17^#^	0.22^#^	0.15^$^	1.00				
U-Alb	−0.02	−0.02	0.03	−0.16^$^	−0.01	0.02	−0.001	0.26*	1.00			
U-A1M	−0.08	−0.03	0.09	−0.003	0.14^$^	0.05	0.12	0.43*	−0.01	1.00		
U-CC16	0.05	−0.05	−0.08	−0.14	0.11	0.001	0.07	0.34*	−0.04	0.57*	1.00	
U-β_2_-m	−0.13^$^	−0.11	0.09	0.01	0.05	0.08	0.06	0.43*	0.08	0.54*	0.28*	1.00

For the units of biomarkers, see Tables 1 and 2. All parameters except age were log transformed. ^$^< 0.05 ^#^< 0.01 *< 0.001

As shown in Table 3, the adjustment on the basis of U-SG apparently abolished the influence of diuresis on U-Cd and LMW proteins since the SG-adjusted values of these biomarkers showed no residual correlation with U-SG. However, one should not infer from this finding that the adjustment with U-SG better corrects for variations in diuresis than the adjustment based on U-Creat. Actually, in some cases, it might be the opposite as the SG-adjusted concentrations of U-RBP, U-Cd, U-Zn and U-Pb showed strong positive correlations with U-Creat (Table 3). Such residual associations were not seen or were less strong when correlating biomarkers per g creatinine with U-SG (Table 3). Of note, U-RBP correlated positively with both U-Zn and U-Pb after adjustment with U-SG but not when expressed per g creatinine, which is the consequence of this under-adjustment with U-SG.

**Fig. 2:**
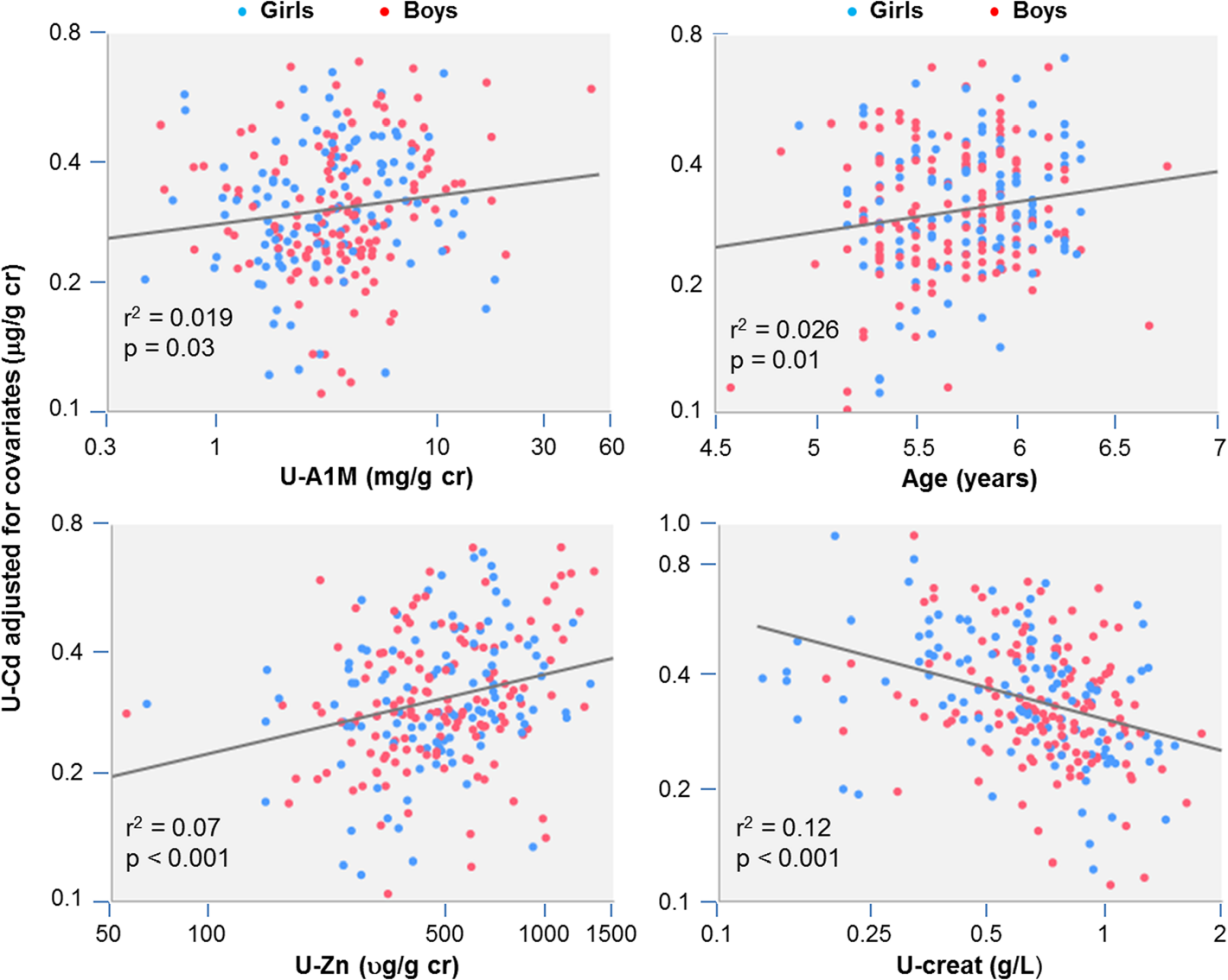
Associations of U-Cd expressed par g of creatinine with U-A1M, age, U-Zn and U-Creat after adjustment for the respective covariates. Adjustments were made using the regression coefficients in Table 4 in the U-A1M model that included U-Creat among independent variables

Determinants of U-Cd were identified by multiple regression analyses testing as potential predictors gender, age, BMI, parental smoking, U-Zn, U-Alb and, in separate models, U-A1M, U-RBP, U-β_2_m or U-CC16. We run these four models by testing five methods of adjustment for urine dilution: 1) U-Cd per g of creatinine, 2) U-Cd per g of creatinine with U-Creat added as separate independent variable 3) U-Cd per liter with U-Creat added as separate independent variable 4) U-Cd adjusted on the basis of U-SG and 5) U-Cd per liter with U-SG added as separate independent variable. As shown in Table 4, in models expressing U-Cd per g of creatinine without any further adjustment, U-Zn emerged as the strongest predictor of U-Cd. Among LMW proteins, it is U-A1M that correlated the most strongly with U-Cd followed by U-CC16, U-RBP and U-β_2_m. Age was retained in all models except in the models run with U-CC16. When further adjusting U-Cd for the residual negative correlation with U-Creat, U-Creat and U-Zn consistently emerged as the main determinants of U-Cd. With this additional adjustment, associations with age, if anything, were strengthened while associations with proteins were weakened, U-A1M and U-RBP being the only LMW proteins retained in the models. The same associations were observed when running these models with U-Cd and other urinary analytes expressed per liter and with U-Creat added to independent variables (Table 4). Figure 2 illustrates the influence of these determinants on U-Cd in the A1M model that best explains the variance of U-Cd. We observed similar patterns of associations in the SG-based models, whether adjusting all urinary concentrations with U-SG or adding U-SG to independent variables and expressing urinary concentrations per liter (Table 5). Virtually the same associations were also observed with U-Cd adjusted with U-SG or with U-Creat and in both cases by adding U-SG or U-Cd to independent urinary variables expressed per liter (results not shown).

**Table 4:** Determinants of U-Cd in models based on U-Creat adjustment

	U-Cd (μg/g creatinine)				U-Cd (μg/g creatinine) with U-Creat as predictor				U-Cd (μg/l) with U-Creat as predictor			
Model	Ind. variable	Regression coefficient (95% IC)	*P*	*r* ^*2*^	Ind. variable	Regression coefficient (95% I)	*P*	*r* ^*2*^	Ind. variable	Regression coefficient (95% I)	*P*	*r* ^*2*^
With U-A1M	U-Zn	0.21 (0.12 to 0.31)	<0.001	0.12	U-Zn	0.19 (0.10 to 0.28)	<0.001	0.23	U-Creat	0.45 (0.30 to 0.60)	<0.001	0.51
(*n* = 249)	U-A1M	0.10 (0.04 to 0.17)	0.002		U-Creat	−0.28 (−0.37 to −0.18)	<0.001		U-Zn	0.18 (0.09 to 0.27)	<0.001	
	Age	0.07 (0.01 to 0.13)	0.03		Age	0.07 (0.02 to 0.13)	0.01		Age	0.08 (0.02 to 0.13)	0.01	
					U-A1M	0.07 (0.005 to 0.13)	0.040		U-A1M	0.07 (0.001 to 0.13)	0.046	
					U-Alb	−0.03 (−0.07 to 0.003)	0.07		U-Alb	−0.03 (−0.07 to 0.005)	0.09	
									U-Pb	0.06 (−0.02 to 0.13)	0.15	
With U-RBP	U-Zn	0.21 (0.12 to 0.31)	<0.001	0.10	U-Zn	0.18 (0.09 to 0.27)	<0.001	0.22	U-Creat	0.44 (0.28 to 0.60)	<0.001	0.50
(*n* = 249)	Age	0.06 (−0.002 to 0.12)	0.06		U-Creat	−0.29 (−0.39 to −0.20)	<0.001		U-Zn	0.17 (0.08 to 0.26)	<0.001	
	U-RBP	0.08 (−0.02 to 0.17)	0.12		U-Alb	−0.04 (−0.08 to −0.01)	0.02		Age	0.07 (0.02 to 0.13)	0.01	
					Age	0.07 (0.01 to 0.13)	0.02		U-Alb	−0.04 (−0.08 to −0.004)	0.03	
					U-RBP	0.08 (−0.01 to 0.17)	0.08		U-RBP	0.08 (−0.01 to 0.17)	0.09	
									U-Pb	0.06 (−0.01 to 0.17)	0.13	
With U-β_2_-m	U-Zn	0.20 (0.10 to 0.30)	<0.001	0.11	U-Zn	0.19 (0.09 to 0.28)	<0.001	0.21	U-Creat	0.56 (0.43 to 0.64)	<0.001	0.51
(*n* = 238)	Age	0.06 (0.007 to 0.13)	0.03		U-Creat	−0.29 (−0.39 to −0.19)	<0.001		U-Zn	0.18 (0.09 to 0.27)	<0.001	
	U-β_2_m	0.06 (−0.01 to 0.12)	0.10		Age	0.07 (0.01 to 0.13)	0.02		Age	0.07 (0.01 to 0.13)	0.02	
	U-Pb	0.06 (−0.02 to 0.15)	0.14		U-Alb	−0.04 (−0.08 to −0.0001)	0.049		U-Alb	−0.04 (−0.07 to 0.001)	0.049	
With U-CC16	U-Zn	0.18 (0.07 to 0.29)	0.002	0.10	U-Creat	−0.31 (−0.44 to −0.20)	<0.001	0.20	U-Creat	0.45 (0.29 to 0.61)	<0.001	0.44
(*n* = 182)	U-CC16	0.05 (0.004 to 0.10)	0.02		U-Zn	0.17 (0.06 to 0.27)	<0.001		U-Zn	0.17 (0.06 to 0.27)	0.002	
	U-Pb	0.07 (−0.02 to 0.16)	0.10		U-Pb	0.06 (−0.02 to 0.15)	0.13		U-Pb	0.06 (−0.02 to 0.14)	0.13	

All parameters except age were log transformed. Independent variables measured in urine were expressed in the same units as U-Cd. For models built with U-Creat in μg/g creatinine without or with U-Creat among predictors, the highest variance inflation factors (VIF) were 1.064 (U-Creat) in the model with U-A1M, 1.058 (U-Alb) in the model with U-RBP, 1.028 (U-Creat) in the model with U-β_2_-m and 1.005 (U-Zn and U-Creat) in the model with U-CC16. For the models built with U-Cd in μg/l with U-Creat among predictors, the highest VIF values were 2.47 (U-Creat) in the model with U-A1M, 2.76 (U-Creat) in the model with U-RBP, 2.26 (U-Creat) in the model with U-β_2_-m and 2.10 (U-Creat) in the model with U-CC16

**Table 5:** Determinants of U-Cd in models based on U-SG adjustment

		U-Cd (μg/l) adjusted to U-SG				U-Cd (μg/l) with U-SG as predictor			
Model	n	Ind. variable	Regression coefficient (95% CI)	*P*	r^2^	Ind. variable	Regression coefficient (95% CI)	*P*	r^2^
With U-A1M	249	U-Zn	0.20 (0.12 to 0.28)	<0.001	0.13	U-SG	34.8 (23.5 to 46.1)	<0.001	0.51
		Age	0.07 (0.02 to 0.13)	0.01		U-Zn	0.20 (0.11 to 0.29)	<0.001	
		U-A1M	0.07 (0.01 to 0.13)	0.03		Age	0.08 (0.02 to 0.13)	0.008	
						U-A1M	0.07 (0.01 to 0.14)	0.02	
						U-Pb	0.06 (−0.01 to 0.14)	0.10	
With U-RBP	249	U-Zn	0.19 (0.10 to 0.27)	<0.001	0.12	U-SG	34.0 (22.5 to 45.5)	<0.001	0.51
		Age	0.07 (0.02 to 0.13)	0.01		U-Zn	0.18 (0.09 to 0.27)	<0.001	
		U-RBP	0.08 (−0.002 to 0.16)	0.06		Age	0.07 (0.02 to 0.13)	0.01	
						U-RBP	0.09 (0.01 to 0.18)	0.02	
						U-Pb	0.06 (−0.01 to 0.14)	0.10	
With U- β_2_-m	238	U-Zn	0.20 (0.12 to 0.29)	<0.001	0.11	U-SG	39.4 (28.1 to 50.6)	<0.001	0.50
		Age	0.07 (0.01 to 0.12)	0.02		U-Zn	0.20 (0.11 to 0.29)	<0.001	
						Age	0.07 (0.01 to 0.13)	0.02	
						U-Pb	0.07 (−0.001 to 0.14)	0.09	
With U-CC16	182	U-Zn	0.17 (0.06 to 0.27)	<0.001	0.09	U-SG	30.4 (17.4 to 43.4)	<0.001	0.42
		U-Pb	0.08 (0.003 to 0.17)	0.042		U-Zn	0.19 (0.09 to 0.30)	<0.001	
						U-Pb	0.09 (0.006 to 0.17)	0.04	

All parameters except age were log transformed. Independent variables measured in urine were expressed in the same units as U-Cd. For models built with variables adjusted to U-SG, the highest variance inflation factors (VIF) were 1.001 (U-A1M) in the model with U-A1M, 1.053 (U-Zn) in the model with U-RBP, 1.001 (U-Zn and age) in the model with U-β_2_-m and 1.005 (U-Zn and U-CC16) in the model with U-CC16. For the models built with U-Cd in μg/l with U-SG among predictors, the highest VIF values were 1.98 (U-SG) in the model with U-A1M, 2.05 (U-SG) in the model with U-RBP, 1.86 (U-SG) in the model with U-β_2_-m and 1.85 (U-SG) in the model with U-CC16. Contrarily to what is observed with U-Creat, there is no residual correlation between SG-adjusted U-Cd and U-SG, which explains that adding U-SG among independent variables does not change the models based on variables adjusted U-SG

**Fig. 3:**
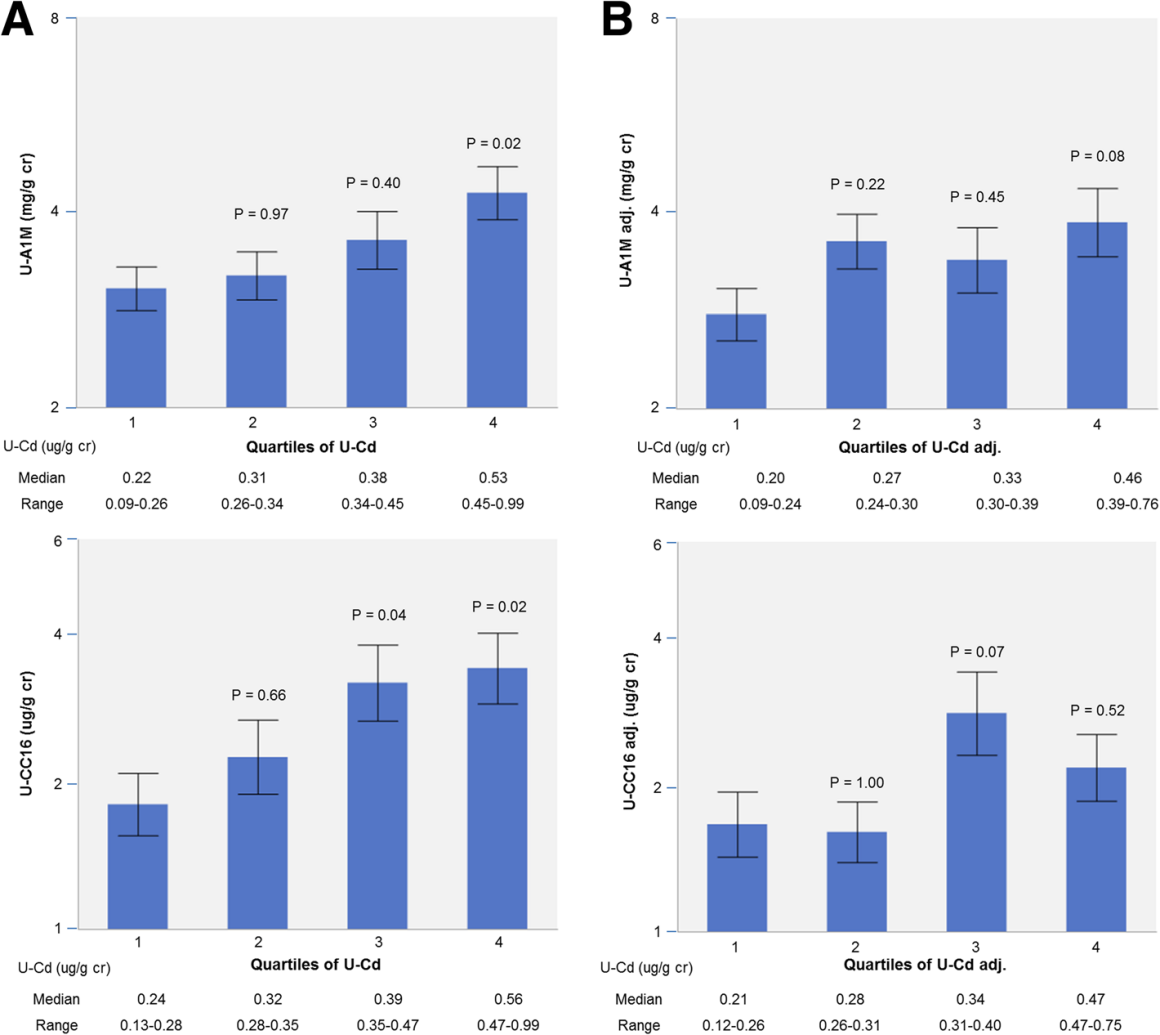
Relationships of U-A1M and U-CC16 with quartiles of creatinine-adjusted U-Cd before (panel **a**) and after (panel **b**) adjustment for the residual associations of these biomarkers with U-Creat. U-Cd adj., U-A1M adj. And U-CC16 adj. Refer to the adjustment based on the univariate regression coefficient of the creatinine-adjusted values of these biomarkers with U-Creat. One-way ANOVA for U-A1M and U-CC6: panel **a**, *P* = 0.04 and 0.02; panel **b**, *p* = 0.17 and 0.06, respectively. The *P*-values in the Figure refer to the Dunett’s post hoc test using the first quartile as control group

We completed our analyses by examining to what extent the creatinine over-adjustment of U-Cd and U-LMW proteins can be a source of confounding when using these biomarkers to assess renal effects of Cd. As shown in Fig. 3, expressed per g creatinine, U-A1M and U-CC16 increase dose-dependently across quartiles of U-Cd, reaching the level of statistical significance from a median U-Cd of 0.53 and 0.39 μg/g creatinine, respectively (ANOVA, *P* = 0.04 and 0.02). After further adjusting these biomarkers for their residual univariate correlation with U-Creat, these dose-response relationships lose their statistical significance (ANOVA, *P* = 0.17 and 0.06, respectively). These relationships were similarly abolished when further adjusting U-Cd for U-Zn and its other covariates (Table 4) (ANOVA, *P* = 0.14 and 0.31, respectively).

## Discussion

Researchers on environmental health increasingly utilize urinary biomarkers to characterize exposures. Associations between chronic diseases and biomarker levels can be interpreted as possibly causal on the condition that the amount of chemical found in urine accurately reflects the long term exposure to the toxic substance under study. It is also important to ensure that the level of biomarker is not influenced by studied outcomes, in which case this would be a source of spurious associations. All these issues are especially critical for U-Cd, a biomarker that most epidemiologists and regulatory bodies rely on to assess lifetime exposure to the metal. In addition, as Cd primarily targets the kidney, there is the challenge of distinguishing associations of U-Cd with renal biomarkers that are caused by Cd nephrotoxicity from associations that reflect the influence of renal function on the excretion of the metal [11, 27, 28].

Regarding the physiological confounders of U-Cd, our study confirms that the concentrations of U-Cd are substantially altered by the method used for urine concentration adjustment [28, 29]. Expressed per liter, U-Cd shows a strong positive correlation with U-Creat, which makes indispensable an adjustment for the hydration status. However, as previously reported [12, 17, 28], this positive correlation turns into a negative one when U-Cd is expressed per g creatinine. This means that dividing the concentration of U-Cd by that of creatinine, as systematically done in most studies, does not completely abolish the influence of diuresis but simply reverses its direction. The important new finding made in our study is that such a correlation inversion also occurs with LMW proteins at the exception of U-RBP. Associations of U-A1M, U-CC16 and U-β_2_m with U-Creat, initially positive, also turned negative when expressing the concentrations per g creatinine. This phenomenon is a source of confounding as it links U-Cd to LMW urinary proteins, in particular A1M and U-CC16, through secondary associations due to physiological variations unrelated to Cd nephrotoxicity. The risk of confounding is especially high, as these associations resemble those induced by high Cd exposure, presenting a U-Cd threshold above which LMW proteins increase in a dose-dependent manner. As almost all studies on the renal effects of Cd were based on Cd and LMW proteins in urine expressed per g of creatinine, this raises the question to what extent associations reported in these studies were distorted if not generated by physiological variations in diuresis. Of course this is especially relevant for associations with low U-Cd but the possibility of a dose-response relationship distortion at high doses of Cd cannot be excluded. Adjusting for U-SG does not appear to be the ideal alternative since in that case U-Cd remained positively associated with U-Creat, testifying to an insufficient adjustment for urine dilution. Different methods can be used to avoid confounding by diuresis. When U-Cd is expressed per g of creatinine, the residual association with U-Creat can be eliminated by further adjusting U-Cd to creatinine on the basis the regression coefficient between the two variables. Currently, the most recommended method is that of Barr et al. [26] in which U-Cd is expressed per liter and adjusted with creatinine on the basis of the regression coefficient between the two analytes. For multiple regression analysis of population groups, this can also be done by including U-Cd and U-Creat, expressed per liter, among independent variables, which allows to build a model in which associations are independent of the effects of urine concentration.

Our study provides further insight into the mechanisms underlying the co-excretion of U-Cd with urinary proteins as described in recent studies [14, 15, 17]. In essence, this mechanism relies on the fact that Cd is excreted in urine as a complex with metallothionein (Mt), a LMW protein that follows the same glomerular filtration-tubular reabsorption pathway as other proteins, including the LMW proteins used for screening Cd nephrotoxicity [30]. We previously hypothesized that the associations of low-level U-Cd with LMW proteins were the reflection of the physiological variations in the protein reabsorption capacity of proximal tubules [14]. The present study demonstrates that, as suggested by Akerstrom et al. [15, 16], for some proteins, this co-excretion is to a large extent driven by variations in urinary flow as estimated by U-Creat. In multiple regression analyses, introducing U-Creat among independent variables noticeably weakened the associations of U-Cd with U-A1M while that with U-CC16 lost its statistical significance. In univariate analyses, also, further adjusting U-Cd for the residual association with U-Creat abolished the dose-dependent increase of U-A1M and U-CC16 with increasing U-Cd. Another mechanism that we postulated is a competitive inhibition of the tubular reabsorption of Cd-metallothionein (Cd-Mt) by filtered plasma proteins. Such a mechanism might explain why associations of U-Cd are much stronger with U-A1M and U-CC16 than with U-RBP and U-β_2_m. The reabsorption of proteins by the proximal tubule is indeed a high capacity, low affinity and saturable process in which proteins compete with each other according to their affinity for the tubular binding sites (mainly determined by their net positive charge) and their relative concentration in tubular fluid [14]. The concentration of A1M in tubular fluid is approximately three orders of magnitude higher than that of CC16 or Cd-Mt. against about only one order of magnitude higher than that of RBP or β_2_m. Because of these huge differences in concentrations, CC16 and Cd-Mt. are conceivably much more easily displaced from tubular binding sites by A1M than are RBP and β_2_m. In other words, the correlation of U-Cd with U-A1M and U-CC16 would be the consequence of the competitive inhibition of CC16 and Cd-Mt. reabsorption by high filtered load of A1M.

Among determinants of U-Cd unrelated to the renal function, we identified U-Zn as the most significant predictor. This association, reported in the adult general population in Japan [31, 32], is not really surprising as the two metals are frequently associated in foodstuffs and also share the same intestinal transporters [33, 34]. Unlike the co-excretion of Cd with proteins, that between U-Cd with U-Zn does not seem to be driven by common renal excretion mechanisms. Although Cd-Mt. transports some Zn, the proportion of U-Zn bound to this protein in urine is much too low to explain this co-excretion. In addition, we found no correlation between U-Zn and urinary proteins, including albumin, which is the main Zn-transporting protein in plasma. The explanation for the co-excretion of the Zn and Cd might thus lie in the homeostatic regulation of Zn intestinal transporters that are opportunistically used by Cd. Previous studies, indeed, have shown that Zn intake or serum Zn correlates negatively with the concentrations of Cd in blood or urine, presumably because of a down-regulation of the intestinal Zn transporters at high Zn intake [35, 36]. The positive correlation between U-Cd and U-Zn seen in our study might be explained by the opposite effect i.e. an up-regulation of the intestinal Zn transporters to meet the important Zn needs of growing children. This explanation might also hold for the positive correlation between U-Cd and U-Zn observed in Japanese populations whose Zn requirements are not completely satisfied by rice, a staple food poor in Zn. Because Zn is an essential nutrient for child growth and development, associations between U-Cd and outcomes such as retarded growth or developmental outcomes should be interpreted with caution [6–9]. These associations might well be secondary to the up-regulation of Zn transporters to meet Zn requirements of the growing child, especially when they are found in poorly nourished children subsisting mainly on rice [8, 9].

Despite the narrow age range of our children, U-Cd was weakly but consistently associated with age. Traditionally, this increase of U-Cd with age is interpreted as the evidence that U-Cd reflects the accumulation of the metal in the body. Assuming that this is the case, it is clear that the contribution of Cd body burden to the U-Cd of children is completely blunted by the influence of other covariates. The U-Cd of our children was indeed similar and when adjusted for U-creatinine even higher than values we recently found in middle age adults in Belgium, despite a Cd body burden at least a five times lower [37]. Of interest, in very young children, U-Cd was not influenced by gender, body mass index or passive exposure to tobacco smoke. There were also no gender-differences in the residual associations of U-Creat with creatinine-adjusted values of urinary Cd and LMW proteins. The only exception concerned U-A1M for which the residual association with U-Creat was much stronger in girls than in boys. The reason for such difference is unknown but it would be interesting to determine if this potential source of confounding is relevant for adults as according some studies U-A1M might be a more sensitive indicator of Cd nephrotoxicity than U-RBP or U-β_2_m [38, 39].

In addition to the risk of confounding by physiological determinants of U-Cd, there is also a risk a misinterpretation or misclassification due to analytical biases. The accuracy of our U-Cd measurements was ascertained by the results of our participation to external quality assurance programs that showed a very good compliance with reference values. Our values of U-Cd (median, girls, 0.22 μg/L; boys, 0.24 μg/L) were almost identical to values in Belgian adolescents reported by us (mean age, 15.4 years; median, girls, 0.27 μg/L and boys, 0.24 μg/L) or by Vryens et al. [40] (mean age, 14.8 years; geometric mean, 0.24 μg/L). Similar values were observed in children living in industrial areas in southwestern Spain (geometric mean, 0.22 μg/L) [7]. By contrast, these values in Belgium and Spain were about 4 times higher than those found in children of the COPHES/DEMOPHES European project (5–11 years, geometric mean of U-Cd adjusted for age, gender and U-Creat, 0.071 μg/L) [41]. Quite surprisingly, in the European project, values of U-Cd for Belgian children were approximately 4 times lower than our values when expressed per liter (0.05 vs. 0.23) and almost 7 times lower when expressed per g of creatinine (0.05 vs. 0.34). By contrast, the mothers of these children had U-Cd values (median, 0.22 μg/L) comparable to values we reported for Belgian adults (median, 0.28 μg/L) if one takes into account that the proportion of current smokers was higher in our study than in the Belgian cohort of the European project (24.1% vs. 9.3%). It should be noted that in the COPHES/DEMOCOPHES project the median U-Cd values of children in Western Europe varied widely by a factor up to seven when comparing Denmark (0.024 μg/L) with United Kingdom (0.167 μg/L) [42]. Even between two small border countries like Belgium and Luxembourg, median U-Cd levels of children differed by a factor of three (0.046 and 0.154 μg/L, respectively) despite very similar U-Cd values for their mothers (0.224 μg/L and 0.249 μg/L, respectively). Furthermore, according to the COPHES/DEMOCOPHES project, Belgian children would be among the less exposed to Cd in Europe, which is astonishing given the important historical pollution of Belgium by non-ferrous smelters. As Cd analyses in the European project were performed by 15 different laboratories, we think that these inconsistencies in the U-Cd values of European children are more likely to be explained by an insufficient analytical harmonization than by true differences in Cd exposure related to the environment or nutritional status.

## Conclusions

The strongest determinants of U-Cd expressed per g creatinine or adjusted to SG, are U-Zn, age and LMW proteins in urine, especially A1M and CC16. The adjustment for urine dilution with creatinine, but not with SG, linked U-Cd to U-A1M or U-CC16 through secondary associations that may be confused with those induced by Cd nephrotoxicity. These physiological influences on U-Cd of young children might confound the renal and developmental effects seen at low-level U Cd.

## Abbreviations

BMI: body mass index
Cd-Mt.: cadmium-metallothionein
IQR: interquartile range
LMW: low molecular weight
U-A1M: alpha_1_-microglobulin in urine
U-Alb: albumin in urine
U-CC16: club cell protein in urine
U-Cd: cadmium in urine
U-Creat: creatinine in urine
U-RBP: retinol-binding protein
U-SG: specific gravity of urine
U-Zn: zinc in urine
U-β_2_m: β_2_-microglobulin in urine
